# A Review of Current Analgesic Techniques in Cardiac Surgery. Is Epidural Worth it?

**DOI:** 10.15171/jcvtr.2014.001

**Published:** 2014-09-30

**Authors:** Mohsen Ziyaeifard, Rasoul Azarfarin, Samad EJ Golzari

**Affiliations:** ^1^Rajaie Cardiovascular, Medical and Research Center, Tehran, Iran; ^2^Department of Anesthesiology and Intensive Care, Tabriz University of Medical Sciences, Tabriz, Iran

**Keywords:** Analgesia, Thoracic Epidural Anesthesia, Cardiac Surgery, Postoperative Pain, Review, Risks

## Abstract

In this review we addressed the various analgesic techniques in cardiac surgery, especially regional methods such as thoracic epidural anesthesia (TEA). There are many techniques available for management of postoperative pain after cardiac operation including intravenous administration of analgesic drugs, infiltration of local anesthetics, nerve blocks, and neuroaxial techniques. Although there are many evidences declaring the benefits of neuroaxial blockade in improving postoperative well-being and quality of care in these patients, some studies have revealed limited effect of TEA on overall morbidity and mortality after cardiac surgery. On the other hand, some investigators have raised the concern about epidural hematoma in altered coagulation and risks of infection and local anesthetics toxicity during and after cardiac procedures. In present review, we tried to discuss the most recent arguments in the field of this controversial issue. The final conclusion about either using regional anesthesia in cardiac surgery or not has been assigned to the readers.

## Pain and Cardiac Surgery

Traumatic or surgical damage induces modifications in the nervous system (peripheral and central) and should be considered during decision making throughout providing analgesia. The possible benefits of reducing the peri-operative stress response are still controversial. However, it is obvious that insufficient post-operative pain control, an uncontrolled surgical stress response, could initiate pathophysiologic alterations in all major organs that may cause considerable postoperative morbidity.^1-3^

Satisfaction of patients is usually correlated to the comparison with expected and experienced pain instead of the actual level of experienced pain. Satisfaction is reached when a condition is better than anticipated. Patients undergoing cardiovascular operation are very worried about post-operative pain and have the tendency to anticipate more post-operative pain than actually experienced.^4,5^ Due to these special pre-operative expectations, patients who post-operatively suffer only moderate analgesia will probably still be pleased with their pain treatment. Thus, patients may have moderate post-operative pain yet still express higher satisfaction levels.^6,7^ In a meta-analysis, Liu et al.^8^ evaluated effects of peri-operative neuraxial analgesia on outcome after CABG. Thoracic epidural blockade did not reduce mortality or MI; however, it appeared to decrease the risk of arrhythmias (AF and tachycardia), respiratory complications (atelectasis and pneumonia), tracheal intubation time, and visual analogue pain scores.^9^

Indeed, intrathecal analgesia did not affect frequencies of mortality, MI, dysrhythmias, or endotracheal intubation time and appeared only to modestly reduce systemic opioid usage and pain scores (while rising the occurrence of pruritus). These authors suggested that neuraxial block methods do not affect the rates of mortality or MI after CABG surgery yet may be related with improvements in weaning from mechanical ventilation and earlier tracheal extubation, decreased respiratory complications and arrhythmias, and decreased pain scores.^8^ However, most possible clinical profits (earlier extubation, reduced arrhythmias, and improved analgesia) of these methods may be attained by other ways, such as the use of fast-track anesthesia protocols, beta blockers or amiodarone, and non-opioid analgesic agents. When critically appraised, the literature advocates that these techniques consistently provide improved postoperative analgesia with no clinically significant effect on mortality and morbidity.^9^

Karagoz et al. reported 5 patients undergoing single-vessel CABG through mini-thoracotomy using only high TEA while completely awake with spontaneous breathing.^10^ TEA can be an alternative option to decrease the stress response of CAGB by eliminating the need for general anesthesia and also mechanical ventilation.^11,12^ Blockade of sympathetic nervous system in perioperative period can decrease post-operative myocardial ischemia. This effect is mainly due to myocardial oxygen demand-supply balance. TEA could reduce the secretion of epinephrine and create this balance inside the autonomous nervous system. TEA, reducing HR and maintaining BP in normal range in patients with insignificant coronary artery disease, may increase oxygen supply and moderate the demand. These effects can enable beating heart CABG surgery to administer lower HR and acceptable BP.^13^

## Pain management modalities after cardiac surgery

Surgical or painful injuries initiate alteration in the nervous system that promote patho-physiologic transformation in major organs; this could lead to extensive postoperative morbidity.^14^ Postoperative pain in cardiac surgery might be severe and may originate from many causes including the surgical incision, intraoperative tissue stress response and dissect of tissue, location of vessels cannulation, chest tubes and venues harvest sites.^15^ Patients in whom internal mammary artery is used for graft may experience significant pain after surgery.^6^ Another frequent origin of pain in patients after cardiac surgery is chest rib fractures. The patients’ age also influences pain strength; patients less than 60 years report more intense pain than older patients.^16^ Constant pain after heart surgery, although rare, can be challenging.^17^ The cause of constant pain after cardiac surgery is multifactorial.^18^ Tissue damage, scar creation, rib fracture, sternal wound infection, intercostal nerve distress, costo-chondral disconnection, and loss of steel wire sutures may all play roles.^10,19^

### Postoperative Analgesia

Pain management has been transferred from intraoperative into perioperative period throughout the emergence of modern anesthesiology. Pain management in postoperative period is one of the most essential components of sufficient post-surgical patients care.^20,21^ Insufficient analgesia in the postoperative period might lead to many unfavorable problems, hemodynamic instability (hypertension, tachycardia and vasoconstriction), immunologic disturbance (impaired immune response), metabolic (extensive catabolism), and hemostatic disorder (platelet activation).^22,23^ Intraoperative surgery and start of cardiopulmonary bypass (CPB) cause extensive secretion of stress reaction hormones (epinephrine, norepinephrine, etc.) that continue to the immediate post-operative period and may lead to myocardial ischemia in this period.^20,24-26^ ,After surgery, myocardial damage might be provoked by cardiac sympathetic nerve reactions which disrupt the stability among coronary blood stream and myocardial oxygen demand.^27,28^ Therefore, during the post-operative period, sufficient analgesia may potentially reduce morbidity and improve quality of life.^29^ Management of postoperative pain could helpfully influence outcome in patients undergoing cardiac surgery.^10,20,28,30^

There are many techniques available for management of postoperative pain after cardiac operation, including:

-Intravenous administration of opioids and non-steroidal anti-inflammatory drugs, alpha-adrenergic drugs;
-Infiltration of local anesthetics;
-Nerve blocks;
-Epidural techniques;
-Intratechal techniques;

All of these techniques have benefits and risks (advantage and disadvantage).^10^

### Intravenous administration

Traditionally, analgesia is provided by opioid analgesic administration after surgery. Nevertheless, extreme opioids administration is associated with a range of side defects including respiratory depression, sedation and lethargy, vomiting and nausea, constipation, urinary retention, purities and ileus.^19,20,31^

Therefore, for superior pain management, physicians use multimodal pain relief regimens such as non-opioid analgesic drugs (local anesthetic drugs, non-steroidal anti-inflammatory drugs(NSAIDS), cyclo-oxygenase inhibitors(COX), *acetaminophen, dexmedetomedine, clonidine, ketamine, gabapentin)* as complement of opioid analgesics that may lead to reduced side effects of opioids.^19,20,32^

Patient control analgesia (PCA) is one of the most common techniques for postoperative pain management. This device is under patient control intermittently or continuously, and infuses IV opioids or non- opioids. Other methods of PCA are patient-controlled epidural analgesia (PCEA) and also patient-controlled regional analgesia (PCRA).^20,33^

### Postoperative local anesthetic infiltration

In the cardiothoracic surgery, thoracotomy or sternotomy incisions are associated with severe pain. These incisions are associated with a decrease in pulmonary function and increased cardiac morbidity.^10,30^ The catheter is inserted at the median sternotomy incision location at the end of surgery for infusion of local anesthetic drugs. This technique seems proficient (improved analgesia, early ambulation, and decreased hospital stay); however, some questions have been raised regarding catheter protection from infection and tissue necrosis.^10,34^ The infiltration catheter techniques result in significant reduction in opioid requirement.^10,24^ Recently, liposomal bupivacaine has been used in infiltration of sternotomy incisions in cardiac surgeries such as robotic or minimal invasive procedures.^35^

### Nerve blocks

Nerve blocks such as intercostal, intra-pleural, and para-vertebral blocks (PVBs) are effective techniques in supplementing other analgesic techniques.^36^

### Paravertebral Block

The PVBs of thoracic segments are utilized in special surgeries both for intraoperative and postoperative pain relief.^22^ The local anesthetic is injected near to the vertebral canal of thoracic region close to where the spinal nerve emerges from the intervertebral foramen. From injection site, local anesthetics drugs transfer both caudally and rostrally.^33,34^ Advantages in these blocks include: simple to learn, secure to present on sedated and respiratory patients, low complication incidence, early mobilization and short hospital stay. PVB are often used unilaterally and can be useful in patients with increased risk of performing neuraxial techniques (coagulopathies). Bilateral PVBs are also used in two-sided breast surgery, vascular and thoracic surgeries.^10,28,33^

### Intercostal block

These blocks are frequently applied for analgesia after cardiothoracic surgery. Intercostal nerve blocks have coupled with extensively decrease supplementary analgesic necessities.^26^ Intercostal nerve blocks could be used both intra-operatively and post-operatively. Local anesthetic drugs may be injected as a single dose or multiple percutaneous doses before chest closure under direct vision, or via an intercostal catheter.^28^ Local anesthetics can provide analgesia and improve respiratory function after cardiothoracic surgery.^24^

### Intrapleural block

An intrapleural catheter placed between the visceral and parietal pleura may provide local anesthetic drugs delivery via bolus or continuous infusion.^28^ Toxicity due to systemic absorption of local anesthetic drugs is frequent with this technique.^26^ A clinical study in which patients underwent thoracic surgery with thoracotomy incision suggests that 0.25-0.5% bupivacaine may improve analgesia in patients following thoracic surgery and also intrapleural block (with 0.25% bupivacaine) is perfect, safe, and provides suitable postoperative analgesia.^24,28^

### Intrathecal analgesia

For the first time in 1980, Mathews and Abrams used intrathecal analgesia in patients undergoing cardiothoracic surgery.^10,22^ The advantages of this technique for analgesia have been explained extensively. The patients remain significantly calm, more cooperative, and able to maintain mobility more easily in bed.^17,22^ Most clinical studies have used intrathecal morphine for providing prolonged analgesia after surgery. Some clinical studies have suggested injecting intrathecal sufentanil, fentanyl and local anesthetics for intraoperative anesthesia and analgesia.^10,17,31^ The most favorable dose of intrathecal morphine to reach the most excellent post-operative analgesia with least unwanted drug side effects is unclear. ^22^

While larger doses of intrathecal morphine are applied, more extreme and lengthened pain relief is created; however, it may be ensued by more frequent unwanted drug adverse effects.^17,31^ Some authors have applied local anesthetics to create a “high-spinal” anesthesia.^22^ This technique is intended for analgesia and sympatholysis but may as well lead to complications such as bradycardia, and hypotension respiratory depression, nausea, vomiting and pruritus^10,31^ Lena and coworkers considered adding clonidine to morphine.^37^ The patients in the clonidine group had improved analgesia and faster extubation time after surgery compared with those with morphine alone.^24^

### Epidural analgesia

Epidural analgesia is effectively applied in some surgeries to improve perioperative pain. In cardiothoracic surgery, epidural analgesia is coupled with improved analgesia, earlier extubation time, better hemodynamics, less respiratory complications, and superior left ventricular function.^22,38,39^ Important complications in this technique are hypotension, epidural abscess, epidural hematoma, and epidural abscess.^10,19,20^ The low incidence of these complications is approving in the obstetrics population and favorable for acute pain.^22^ Epidural hematoma is usually regarded as a really rare incident in non-cardiac surgery. For example, while epidural catheters were applied in obstetrics, the considered risk of epidural hematoma was 1 in 168,000.^10,20,32^ Cardiac surgical patients signify a high risk group for frequent reasons. These include the usage of intra-operative heparin, elder age, and longer period of infusion.^33^ In a study, bupivacaine only, bupivacaine with clonidine and bupivacaine with fentanyl were compared. There was no difference in heart hemodynamics, nausea, vomiting and sedation level. The pain scores were extremely low for all three groups.^22,31^

General anesthesia (GA) is the anesthetic regimen of choice in patients undergoing coronary artery bypass grafting (CABG) and is considered the “gold standard” for these operations performed either on pump or off pump. In the past decade, however, high thoracic epidural anesthesia (TEA), as an alternative or complementary technique to GA, has become more commonly used and reported to be theoretically helpful in patients with coronary artery disease.^40^ Possible benefits of TEA consist of thoracic sympathicolysis with following enhancement of coronary perfusion, decreased heart rate (HR), especially in patients undergoing off pump CABG, reduced endogenous stress response, and a decreased possibility for myocardial ischemia. Furthermore, enhanced gastrointestinal function with simultaneous decline in morbidity and mortality have been described.^41,42^ Better hemodynamic stability and postoperative pulmonary function have also been reported when combined TEA and general anesthesia were used for CABG.^43^ In addition, postoperative pain management is enabled by continuous epidural administration of analgesic agents. This efficient pain management enables postoperative mobilization and recovery.^44^ Side effects of TEA are infection and hematoma formation with consequent adverse neurologic sequelae.^45^

Some meta-analyses and randomized-controlled trials have suggested improved analgesia, fewer respiratory complications, maintained acceptable cardiac function, lower frequency of renal insufficiency, and quicker recovery in comparison with opioid-based methods.^46-48^ Nonetheless, some disagreements exist as these investigations had limited numbers of patients and were occasionally not controlled.^48-50^

## Sympathetic activity and stress response

The augmented sympathetic activity accompanied by injury makes changes in the host’s hormonal and immune response and in the coagulation system.^51^ These defense mechanisms could turn against the host in those with co-existing cardiovascular disease.^52^ Increased plasma catecholamine levels rise heart rate and left ventricular afterload while the time for coronary perfusion is reduced. Stenotic coronary arteries cannot tolerate this sympathetic activation.^52^ After major cardiac surgery, serum levels of stress hormones increases.^51^ This situation induces a pro-coagulatory state in patients with co-existing cardiovascular disease. In conclusion, early after stressful insult, a pro-inflammatory response can lead to plaque instability.^53^ This pathophysiologic cascade initiates acute coronary syndrome and myocardial ischemia during and after stressful procedures. As a result, cardiovascular events account for about two third of mortality cases in a high-risk patients after major surgeries.^54^

## TEA and sympathetic blockade

The extent of segmental sympathetic block in TEA is a main factor of the perioperative effects of TEA. Procedural problems prevent objective evaluation of sympathetic activity in the perioperative period. In other words, there is no tool for direct measurement of sympathetic block in clinic, so anesthesiologists use surrogate methods such as skin temperature for estimating sympathetic block. Indirect methods like skin conductance response and heart rate fluctuation rely on altered function of organ undergoing sympathetic block.^55^ Majority of assessments are based on the evaluation of skin perfusion. Changed skin temperature regulation is revealed by thermography in TEA.^56^ In humans, sympathetic block affects splanchnic and lower limb nerves with limited upper thoracic sensory block with high TEA after injection of bupivacaine. The concentration and dose of the local anesthetic may balance the edges of the sympathetic block.^57^

## Anti-ischemic properties of TEA in cardiac surgery

There are evidences supporting that TEA decreases perioperative cardiac complications.^58^ Superior pain relief with accompanied decrease in the postoperative stress response and systemic sympathetic activity may play a role to this effect. Segmental sympathetic block of the cardiac sympathetic nerves not only decreases ischemic pain but also maintains coronary perfusion. This effect is more significant in stenotic coronary arteries.^59^ TEA enhances ventricular diastolic function in patients with CAD undergoing CABG surgery. Diastolic dysfunction has been shown to be an early sign of cardiac ischemia. A study reported enhanced systolic function and also wall motion in coronary artery disease.^60^ Decreased troponin release and prolonged survival after CABG suggest the cardio-protective effect of TEA. The largest prospective trial of the outcomes of TEA did not display a survival benefit.^61^ Some meta-analyses propose that TEA might reduce cardiac morbidity and mortality after cardiac and major non-cardiac operation. However, other studies have neither proven this effect nor the stress on decreased respiratory complications or arrhythmias after cardiac operations.^54,62^

## Risks of TEA

### Epidural bleeding and hematoma

Neuraxial hematoma is commonly considered as the most thoughtful complication of peridural anesthesia. The risk of paraplegia due to hematoma in the cardiac surgical patients after TEA has been reported to be between at least1:150,000 up to 1:1,500, with a 95% confidence interval (CI).^63,64^ In a report in Sweden, the risk of bleeding into the vertebral canal (in obstetric population) in the 1990s was 1:18 000.^65,66^ The risk of epidural bleeding in a study was greater with incidence of 1:10 200 for surgical patients in peri-operative period.^67^ Freise et al., in a single-center survey in 2011, reported a higher incidence ranging between 1:2700 and 1:4761.^53,68^ However, some investigators have reported lower estimated incidences of hematoma including 1:220,000 after intra-thecal block and about 1:150,000 after epidural blockade.^69^

Epidural hematoma does not happen solely in catheter insertion; nearly half of cases of hematoma formation ensue after catheter removal.^70^ Systemic heparinization increases the risk of bleeding during lumbar puncture.^71^ However, by considering definite precautions, lumbar or thoracic epidural puncture can be done safely in patients who will take IV heparin later.^72^ By postponing the operation for 24 hours in the occasion of a traumatic tap or by postponing heparinization 60 minutes after catheter insertion, and also by keeping tight peri-operative monitoring of anticoagulation, neuroaxial blockade can be done safely.^73^ An extensive mathematical analysis by Ho and colleagues^74^ on 10,840 intra-thecal punctures in patients undergone IV heparinization for CPB (with no episode of hematoma creation) calculated estimation of the lowest risk of hematoma development as 1:220,000, and the highest risk of hematoma as 1:3600 (95% CI).

Majority of studies on patients undergoing cardiac surgery suggest using the technique only after observing laboratory confirmation of normal coagulation values, postpone surgery one day in the occurrence of a traumatic tap, or considering the time from the puncture to systemic administration of heparin after 60 minutes. Some anesthesiologists perform epidural block the day before scheduled cardiac surgery. However, recently some clinicians insert the catheter on the same day of surgery. The instrumentation should not be done in a patient who has known coagulopathy. Also, closed coagulation monitoring should be considered and patients should be carefully observed for postoperative signs and symptoms of bleeding or developing hematoma.^10^

### Side Effects of Local Anesthetics

The most concerning side effect of neuroaxial local anesthetics is hypotension. Upper thoracic spinal anesthesia can decrease mean arterial blood pressure (MAP) that equals reduction in coronary blood flow.^75^ In addition, using alpha adrenergic agents such as phenylephrine increases the arterial blood pressure, but may lead to vasoconstriction in the coronary arteries and also bypass graft conduits.^76^ As hypotension is an essential problem in patients who receive intra-thecal local anesthetics to create a total spinal anesthesia for cardiac surgery, many of them requisite IV phenylephrine use during surgery to rise arterial blood pressure. Hypotension similarly is comparatively common when using local anesthetics through TEA in cardiac surgery. Volume expansion and alpha agonist administration are necessary in most of these patients. As higher doses of local anesthetics are used for TEA, they can reach blood concentrations that may cause harmful cardiac electro-physiologic effects and also lead to myocardial depression.^77^ It is likely that low level thoracic epidural-induced sympathectomy create changes in sympathetic-parasympathetic equilibrium (i.e., vasoconstriction in upper segments of block), producing coronary artery spasm.

### Complications of neuroaxial Opioids

Main adverse effects of epidural and intra-thecal opioids are nausea, vomiting, pruritus, urinary retention, and also respiratory depression.^78^ The most common adverse effect of opioids is pruritus happening in only about 1% of patients. Frequency of nausea and vomiting is more common reaching to around 30%. Occurrence of urinary retention is variable and mostly happens in young male adults. The most fearing side effect of neuroaxial opioids is respiratory depression. The frequency of respiratory depression that needs treatment is about 1%, the similar as that after routine doses of IM or IV opioids. Risk factors of respiratory depression consist of high or repeated doses of narcotics, intra-thecal use, old age, and simultaneous use of IV sedatives. Long post-operative respiratory depression may postpone tracheal extubation, and naloxone may be needed in such situations.^60^

### Infectious Complications

Regarding puncture of physiologic barriers such as skin and dura in TEA, this technique is unavoidably accompanied by the risk of infectious complications. Potential sources of infection within the vertebral canal are iatrogenic micro-organism inoculation and hematogenous infection at the needle insertion site or the catheter.^61^ Incidence of epidural abscess vary widely from 1:10 000 to 1:24 000 patients. Epidural abscess is usually caused by Staphylococcus aureus. Incidence of meningitis is lower and mostly caused by Streptococcus.^79^ Signs of infection commonly are non-specific, however the symptoms may present at the insertion site. This often results in late diagnosis and requires close observation and also a high degree of suspicion. Prognosis of infectious complications is generally better than epidural hematoma.^52^

## TEA and outcome

Although TEA delivers better postoperative pain relief, regardless of better pain control, using TEA appears to be procedure specific and institutional preference. Improvement in quality of early postoperative period could affect long-term psychological status in patients.^80^ Reduction in the rate of respiratory complications after TEA, perhaps reflects earlier embolization, decreased opioid use, and improved airway hygiene. Although some meta-analyses revealed reduction in the occurrence of cardiac complications (MI and arrhythmias), reduced mechanical ventilation time and mild decrease in perioperative mortality with TEA in cardiac surgery patients, recent studies have shown that epidural anesthesia during cardiac surgery using CPB does not affect long-term outcomes significantly.^81^

## Conclusions

Despite the increased popularity of intra-thecal and epidural techniques in cardiac surgery patients, their administration remains very controversial.^81,82^ Main causes for such controversy are the suboptimal clinical investigations, paucity of well-designed researches using dissimilar techniques, and precluding clinically useful conclusions.When critically appraised, the evidence reveals clear advantage of performing intra-thecal or epidural anesthesia in patients undergoing cardiac surgery by better postoperative pain control, while there is no definite evidence of clinically important improvement in outcome.^83^ Although numerous analgesic methods are broadly used, IV narcotics are the keystone of post-cardiac surgery analgesia. The possible benefits obtainable by intra-thecal and epidural techniques consist of strong post-operative analgesia and stress response reduction and also cardiac sympathectomy at thoracic levels. However, there are obvious deficits in the literature that prohibit conclusive exploration of the risk-benefit ratio of neuroaxial techniques for cardiac operation. Upcoming guides for investigation should emphasize on conduct of well-designed researches with sufficient sample size that examine the potential capability of these methods to reduce morbidity (particularly cardiac and respiratory) and mortality after cardiac procedures.^10,84^

## Ethical issues

Not applicable.

## Competing interests

Authors declare no conflict of interest in this study.
